# Depressive symptoms mediate the relationship between cognitive reserve and cognitive performance in middle-aged and older Chinese adults: evidence from population-based and clinical PET cohorts including cognitively normal and cognitively impaired participants

**DOI:** 10.3389/fnagi.2025.1708268

**Published:** 2026-01-05

**Authors:** Yucheng Gu, Xiaoyuan Li, Nihong Chen, Feng Wang

**Affiliations:** 1 Department of Nuclear Medicine, Nanjing First Hospital, Nanjing Medical University, Nanjing, China; 2 Department of Neurology, Nanjing First Hospital, Nanjing Medical University, Nanjing, China

**Keywords:** amyloid, CHARLS, cognitive domains, cognitive reserve, depressive symptoms, mediation analysis, positron emission tomography, tau

## Abstract

**Background:**

Cognitive reserve (CR) may protect cognitive performance under pathology. Depressive symptoms are common in mid and late life and are linked to poorer cognition. This study investigated whether depressive symptoms mediate the association between CR and both global and domain-specific cognitive performance in middle-aged and older Chinese adults.

**Methods:**

Data from 1,636 participants in the 2018 wave of the China Health and Retirement Longitudinal Study (the CHARLS 2018 cohort) were analyzed. Information from an independent retrospective cohort that underwent amyloid and tau positron emission tomography (PET) at Nanjing First Hospital (the PET cohort; *n* = 100) was collected to validate the results from CHARLS. Associations between CR and cognitive performance in memory, executive function, language were examined, and mediation analysis was performed to assess the role of depressive symptoms. Subgroup analyses were also conducted. In the CHARLS 2018 cohort, participants were stratified as cognitively normal or cognitively impaired. In the PET cohort, participants were stratified into amyloid-negative cognitively normal and amyloid-positive mild cognitive impairment (MCI).

**Results:**

Across both cohorts, higher CR was associated with a lower risk of cognitive impairment and better domain-specific performance. Depressive symptoms partially mediated the association between CR and several domains of cognition in the analyses of the overall cohorts. In both the CHARLS 2018 subgroups, no mediation was detected. In the PET cohort, depressive symptoms fully mediated the effects of CR on executive function and attention in the MCI group, in which most participants showed tau deposition on PET, whereas no mediation was observed in the cognitively normal subgroup.

**Conclusion:**

CR is a protective factor for cognition, and depressive symptoms act as a modifiable mediator. In AD patients with confirmed tau pathology, timely detection and management of depressive symptoms may help preserve cognition and enhance the benefits of CR.

## 1 Introduction

Cognitive impairment is a prevalent issue among the middle aged and geriatric population, characterized by a range of symptoms, including memory loss, executive dysfunction, language or visuospatial challenges, and behavioral changes (Anderson, 2019). Mild cognitive impairment (MCI) refers to a decline in cognitive abilities that does not result in significant functional impairment, distinguishing it from dementia (Anderson, 2019), which represents a more severe form of cognitive dysfunction. Over 15% of community-dwelling older adults experience MCI nationally in China (Jia et al., 2020), while the overall prevalence of dementia in China was estimated at 6% (Yin et al., 2024). The large number of patients with cognitive impairment in China underscores the need for enhanced public health initiatives aimed at preventing and managing cognitive impairment, particularly given the country's aging population (Jia et al., 2020).

Demographic influences such as education level, employment status, socioeconomic factors, geographical disparities, and frequency of physical activity are associated with the development of cognitive decline (Jia et al., 2020; Yin et al., 2024; Cong et al., 2023). These demographic variables are also sociobehavioral proxies often used as indirect measures of cognitive reserve (CR), which refers to the brain's capacity to flexibly engage cognitive processes in response to age-related and pathological changes (Stern et al., 2020). A higher CR enables individuals to better cope with cognitive decline (Montemurro et al., 2025; Livingston et al., 2024), allowing them to maintain cognitive function despite underlying brain pathology, thereby acting as a dynamic form of resilience (Stern et al., 2020). Khalaila et al. (2024) suggest that composite CR indices are more comprehensive and sensitive in evaluating cognitive performance, both in global cognition and across specific cognitive domains, compared to using individual CR indicators alone.

Cognitive impairment is increasingly recognized as not confined to late-life but emerging in midlife. Analyses from the Global Burden of Disease 2021 study indicate a global rise in dementia with onset at approximately 30 to 64 years (He et al., 2025; Zhang et al., 2025). China is among the fastest-growing settings, with age-standardized prevalence, mortality, and disability-adjusted life years before age 65 rising steadily from 1990 to 2021. The greatest growth occurred at ages 50 to 64, where prevalence more than doubled (He et al., 2025; Zhang et al., 2025). Findings from the large cohort-based Atherosclerosis Risk in Communities study suggest that midlife (45 to 64 years) is a critical stage for dementia prevention (Gottesman et al., 2017; Knopman et al., 2018). Midlife vascular risk factors and midlife cognitive performance each independently predicted late-life MCI and dementia (He et al., 2025; Zhang et al., 2025), which indicates a cognitive vulnerability pathway in midlife that is not fully explained by vascular mechanisms. It is therefore justified to assess cognitive function and CR in middle-aged adults and to identify those with lower performance at this stage to target early preventive strategies.

The overall prevalence of depression among older adults is approximately 20% in China (Tang et al., 2022), and the lifetime prevalence rates of major depressive disorder (MDD) in China are 1.8% (Zhao et al., 2021). Epidemiological studies have shown a long-term comorbid relationship between MDD and MCI. Among patients with MCI, depression prevalence ranges from 16.9% to 55% (Ma, 2020). Cognitive dysfunction can be commonly observed in older adults with depressive disorders, with approximately one-third meeting the diagnostic criteria for MCI (Marawi et al., 2023). Especially, previous studies indicate that higher CR is associated with better mental health and a lower burden of depressive symptoms, and in some clinical samples with a reduced risk of MDD (Ponsoni et al., 2020; Porricelli et al., 2024; Yang et al., 2024).

Cognitive function comprises multiple domains of cognitive abilities, including memory, perception, orientation, executive function, attention, processing speed, and language skills (Harvey, 2019). With aging or under pathological conditions, in addition to an overall decline in cognitive function, varying degrees of dysfunction across different cognitive domains may also appear (Li et al., 2021). It has been verified that a higher level of CR is associated with improved performance in language fluency, information processing speed, and working memory, and that individuals with high CR tend to have stronger executive function (Panico et al., 2023; Oosterman et al., 2021). Patients with depressive symptoms also exhibit cognitive decline across multiple domains, including impairments in working memory, slowed information processing, difficulties with verbal learning, and deficits in executive function (Venezia et al., 2018; Murphy and O'Leary, 2010).

The China Health and Retirement Longitudinal Study (CHARLS) is a nationally representative cohort study that collects data on sociodemographic characteristics among Chinese adults aged over 45 (https://charls.pku.edu.cn/). Analyses of middle-aged and older adults in the CHARLS, which enrolls participants from age 45 years onward, show that cognitive impairment can be identified in this population and that it is associated with chronic disease and social factors (Zu et al., 2025; Zhang et al., 2025). The mediating role of depressive symptoms between cognitive function and other life course CR-related factors, such as adverse childhood experiences and social participation, was illustrated through analyses of the CHARLS database (Shi et al., 2024; Chen et al., 2024; Wang et al., 2023). On this background, recent authors have proposed that reserve should also include an affective or emotional component that helps individuals regulate stress and mood in the face of aging and neurodegenerative processes (Pinto et al., 2025; Di Rosa, 2024). This emerging notion of affective/emotional reserve is conceptually consistent with the CHARLS findings in which depressive symptoms link reserve-related exposures to cognitive outcomes. By supporting emotional and motivational regulation rather than cognitive efficiency itself, affective/emotional reserve may create a more favorable context for cognition and thereby reduce the impact of depressive symptoms on attention, executive function, and memory (Pinto et al., 2025; Di Rosa, 2024).

Alzheimer's disease (AD) is the most common cause of cognitive impairment, and its core neuropathological features are β-amyloid (Aβ) deposition and tau aggregation (Jack et al., 2024). With current molecular imaging techniques, especially Aβ and tau positron emission tomography (PET), these pathologies can be detected *in vivo* and used to support diagnosis and staging in clinical settings (Jack et al., 2024). CR shows a pathology-dependent effect, with its protective role emerging only beyond certain thresholds of AD pathology (McKenzie et al., 2020). A longitudinal study based on data from the Alzheimer's disease Neuroimaging Initiative (ADNI) found that CR predicted executive function decline in biomarker-positive but not biomarker-negative individuals (McKenzie et al., 2020). Furthermore, CR remains protective even in the presence of marked Aβ deposition, tau elevation, and hippocampal atrophy, helping delay the onset of MCI or dementia (Nelson et al., 2021). Depression is also common among patients with AD, with approximately 38% to 40% of them exhibiting depressive symptoms (Huang et al., 2024). Depressive symptoms are associated with AD pathology, particularly abnormal tau deposition, and this association is observed only in Aβ positive individuals, where depressive symptoms predict more rapid tau progression (Talmasov et al., 2025).

In memory clinics within neurology departments, it is quite common to see patients in their fifties who report memory complaints, meet criteria for mild cognitive impairment, or are suspected to lie on the AD spectrum and are referred for Aβ or tau PET (Xu et al., 2023). Although early-onset AD is conventionally defined as symptom onset before 65 years of age (Reitz et al., 2020), several clinical series have shown that the practical peak of symptom emergence has a mean onset close to 56 years (Koedam et al., 2010; Mendez et al., 2012). Thus, including a clinical PET cohort covering middle-aged and older adults is important in this study. It enables us to test whether the associations among CR, depressive symptoms, and cognitive performance that we observed in the population-based cohort are also present in individuals who are biomarker-defined along the AD spectrum.

Currently, there is still a lack of research on the mediating role of depressive symptoms in the relationship between composite CR and cognitive performance in population-based cohort among middle-aged and older adults, and it remains unknown whether both the mediating effect and the relationship itself may be influenced by AD pathology. Using CHARLS 2018 data, we constructed composite CR indicators and examined associations with cognitive impairment and domain-specific cognition, specifically memory, executive function, and language. Depressive symptoms were then examined as a mediating factor to assess their influence on the relationships between CR and cognitive impairment, as well as between CR and performance in different cognitive domains. To better understand the potential influence of global cognitive status, we aimed to perform subgroup analyses of cognitively impaired and cognitively normal participants. As the CHARLS study did not include neuroimaging data, we retrospectively collected detailed neuropsychological results and AD pathology information from subjects in midlife and late-life who underwent both cognitive tests and PET examinations at Nanjing First Hospital. In particular, the Cognitive Reserve Index Questionnaire (CRIq) (Nucci et al., 2012) was used for composite CR evaluation in these subjects who underwent PET. We conducted parallel analyses in an independent retrospective clinical cohort with PET and other neuroimaging data to validate the CHARLS findings and to further examine the mediating role of depressive symptoms in the association between CR and global and domain-specific cognition across Alzheimer's pathology strata.

## 2 Methods

### 2.1 Study population

#### 2.1.1 CHARLS population-based cohort

Our study utilized publicly available data from the CHARLS wave 2018 (https://charls.charlsdata.com/pages/Data/2018-charls-wave4/zh-cn.html), which was approved by the Biomedical Ethics Review Committee of Peking University (IRB00001052–11015). All participants provided written informed consent prior to participation. As this study involved a secondary analysis of de-identified data, no additional ethical approval was required. The study was conducted in accordance with the ethical standards of the original study protocol. Furthermore, all data used in this research were officially obtained. This current study included all participants aged 50 to 80 years from the 2018 wave of the CHARLS dataset (the CHARLS 2018 cohort). The exclusion criteria included participants with a history of Parkinson's disease or severe mental disorders. In addition, participants with missing data on key demographic or sensory variables (age, sex, self-reported hearing, or self-reported eyesight) were excluded from the analyses. Details of the inclusion and exclusion process are provided in Supplementary Table S1. After exclusion, a total of 1,636 subjects were finally included in the analysis of CR and cognitive impairment. For the further analysis about domain-specific cognitive functions those with information loss in were further excluded and 916 subjects were included at last. Operational definitions of global cognition using Mini-Mental State Examination (MMSE) education-specific cutoffs are summarized here and described in detail in Section 2.2.3.

#### 2.1.2 Clinical PET cohort

For the retrospective cohort from Nanjing First Hospital (PET cohort), information on subjects with normal cognition and AD-originated MCI, aged 50 to 80 years, was collected from the Department of Nuclear Medicine and the Memory Clinic in the Department of Neurology, who underwent PET examinations from September 2023 to June 2025. The Ethics Committee of Nanjing First Hospital approved the study. Inclusion criteria comprised cognitively normal individuals with negative Aβ PET and MCI patients with positive Aβ PET. Some of the Aβ-negative cognitively normal participants and all the Aβ-positive MCI patients additionally underwent tau PET. Among participants who underwent both Aβ and tau PET imaging, all cognitively normal individuals were tau PET–negative, whereas in the MCI group tau PET results were variable, with some positive and some negative. Exclusion criteria included major cerebral infarction, multiple lacunar infarctions, severe white matter hyperintensities (WMH) based on magnetic resonance (MR) imaging findings, parkinsonian symptoms, other causes of cognitive impairment (e.g., hydrocephalus, metabolic disorders), psychiatric disorders (e.g., severe depression, schizophrenia) with long-term administration of sedatives, congenital abnormalities (e.g., Down syndrome, cerebral palsy), and contraindications to MR. To ensure valid administration of neuropsychological tests that are auditory or visually based, participants were required to have normal or corrected-to-normal hearing and vision, and the use of assistive devices (e.g., hearing aids, reading glasses) was permitted. Participants with uncorrected, clinically evaluated moderate-to-severe sensory impairment, or with such impairment combined with very low educational attainment (below middle-school level) that would compromise test validity, were excluded. Ultimately, 50 cognitively normal subjects and 50 MCI patients were included in the study. Details of the inclusion and exclusion process in the PET cohort are shown in Supplementary Table S1. MCI was defined using pre-specified domain-based criteria across memory, executive, and language functions, with full details provided in Section 2.2.3.

### 2.2 Measurements

#### 2.2.1 Cognitive reserve

In both cohorts, CR was conceptualized according to the three domains defined by the CRIq: Education, Working Activity, and Leisure Time (Nucci et al., 2012).

In CHARLS 2018 cohort, survey items were mapped to these domains as shown in Supplementary Table S2, and we constructed a cohort-specific composite to obtain a CR total. Education in CHARLS was available only as the highest educational degree (ordinal), and years of education were not collected, so this ordinal format was retained when constructing the CR composite. The educational level score ranged from 1 to 11 points, from illiteracy to doctoral graduation. The occupational level score ranged from 1 to 5 points, from low-skilled manual work to highly responsible or intellectual occupations. Leisure and social participation items were provided in categorical/binary form in CHARLS, so a “Yes” answer was assigned 1 point and a “No” answer was assigned 0 points. The composite CR in the CHARLS 2018 cohort was calculated as the sum of the scores for education, occupation, and leisure time.

The assessment of CR among the subjects from the PET cohort was based on the CRIq (https://www.cognitivereserveindex.org/). The total Cognitive Reserve Index (CRI) in the PET cohort was calculated following the original CRIq procedure, in which raw scores for Education, Working Activity, and Leisure Time are age-adjusted through linear models, standardized to a scale with mean 100 and standard deviation of 15, and then averaged to obtain the total CRI (Nucci et al., 2012).

Because both cohorts used the same three-domain CR framework but relied on different item formats and levels of scoring granularity, each cohort was analyzed separately and the total CR score in each cohort was dichotomized at the cohort-specific median. Participants with scores above the median were classified as having high CR and those at or below the median were classified as having low CR.

#### 2.2.2 Neuropsychological assessment

In the CHARLS 2018 cohort, global cognition was assessed by the MMSE score (Jia et al., 2021). Among the subjects from the PET cohort, both MMSE and the Beijing version of the Montreal Cognitive Assessment (MoCA) were used to assess global cognition (Yu et al., 2012). The Clinical Dementia Rating sum of boxes (CDR-SB) was used to evaluate the severity of cognitive impairment in the MCI group (Huang et al., 2021).

The neuropsychological assessment in CHARLS 2018 primarily targeted the cognitive domains of memory, language, and executive function. Memory function was further divided into three subsets, namely immediate memory, delayed memory, and recognition memory, and each was analyzed separately. The number of correctly recalled items in each subset was defined as the score for each memory test. Episodic memory was defined as the combined performance on these memory subsets and was calculated as the sum of scores from the three subsets mentioned above. Language function was evaluated through verbal fluency, measured by the total number of correct animal names produced in 60 seconds. Reasoning ability, as a complex cognitive process involving integration, analysis, and conclusion formation, is considered a component of executive function (Burghart et al., 2024) and was tested using a sequence problem. The total number of correct answers was calculated using the Rasch model, and a two-stage adaptive test was employed (Xu et al., 2023). Individuals who answered more items correctly in the first stage received a more difficult test in the second stage.

Accordingly, for the participants from the PET cohort, memory function was assessed using immediate memory, delayed memory, and recognition memory from the Auditory Verbal Learning Test, the Huashan version (AVLT-H) (Zhao et al., 2012). Language function tests were composed of the 30-item Boston Naming Test (BNT) and verbal fluency (Zhao et al., 2012; Guo et al., 2006), whereas executive function tests included the Trail Making Test (TMT) Parts A and B (Wei et al., 2018). We further evaluated attention using the Symbol Digit Modalities Test (SDMT) and both forward and backward Digit Span tests from Wechsler adult intelligence scale-4th edition (Cui et al., 2017). The z-scores for each cognitive domain were calculated based on the sum of the scores from each neuropsychological test and were used to evaluate ability in each cognitive domain. Notably, the z-score for executive function was the negative of its original score to make it positively correlated with cognitive performance.

#### 2.2.3 Cognitive impairment and MCI

In CHARLS 2018, global cognitive status was determined by the MMSE score, with cognitive impairment defined by education-specific MMSE cutoffs. People who were illiterate and scored 19 points or less, those who completed primary school and scored 22 points or less, and those with secondary education or higher who scored 26 points or less were defined as having cognitive impairment (Jia et al., 2021). For the participants from the PET cohort, the global cognitive status was divided into cognitively normal or MCI. MCI was defined according to the operational diagnostic criteria proposed by Bondi et al. (2014) and Edmonds et al. (2015), derived from ADNI data, using results from the AVLT-H, BNT, verbal fluency, and TMT. In brief, a participant was classified as having MCI if they showed impairment on two tests within the same cognitive domain, or impairment on one test in each of three domains: memory, executive function, and language. Cutoff scores for the domain-specific cognitive tests used to define MCI, stratified by age and educational level, are presented in Supplementary Table S3 and follow the operational protocol from a large authoritative Chinese cohort study about cognitive decline (Li et al., 2019; Yu et al., 2024).

#### 2.2.4 Depressive symptoms

The incidence of depressive symptoms among participants in CHARLS 2018 was defined using the 10-items Center for Epidemiologic Studies Depression Scale (CESD-10). The total CESD-10 score ranged from 0 to 30, a total score greater than 10 was defined as the presence of depressive symptoms (Yang and Hou, 2024). In the PET cohort, depressive symptoms were assessed with the 17-item Hamilton Depression Rating Scale (HAMD-17), and scores >7 were considered clinically significant (Zimmerman et al., 2013). In this manuscript, “depressive symptoms” refers to symptom severity derived from these standardized rating scales. We did not collect clinician-based diagnostic information, so study variables are reported as depressive symptoms, and the term “depression” is reserved only when citing studies that examined clinician-diagnosed depression.

### 2.3 Participant characteristics and covariate strategy

The baseline demographics and potential confounders between cognitively normal and cognitively impaired groups were compared in both cohorts. In CHARLS 2018, the following variables were examined: (1) Demographic characteristics, including gender, age, educational level, and marital status; (2) Factors associated with cognitive impairment according to previous studies, including self-reported hearing, self-reported eyesight, residence, and sleeping hours (Bucholc et al., 2022; Ehrlich, 2024; Kramer et al., 2025; Overton et al., 2025); (3) Other self-estimated information, including self-reported health status and self-reported memory status. These variables were used for baseline description rather than being automatically entered into every model. For multivariable analyses in CHARLS 2018, only variables that met our prespecified covariate selection rule were entered, and observations with missing values in those selected covariates were handled using a complete-case approach. We reclassified age into 50–64 years (midlife), 65–74 years (young-old), and ≥75 years (old-old) to reflect distinct life-stage groups and examined between-group differences across these strata, following common epidemiological practice (Gottesman et al., 2017; Knopman et al., 2018; Min et al., 2023).

In the PET cohort, all participants had normal or corrected-to-normal hearing and vision and lived in urban areas. Because sleep and other self-reported lifestyle measures were not collected, baseline comparisons were therefore limited to key demographics, namely age, sex, and years of education.

Age and sex were included a priori in all models. Other demographics and potential confounders were considered for adjustment if they showed meaningful or statistically significant baseline differences between cognitively normal and cognitively impaired groups, or if they were judged to be clinically or causally relevant. When CR was modeled as the primary exposure, education was not included as a covariate because it is embedded in the CR construct and additional adjustment would double count and risk overadjustment and multicollinearity. This rule was applied in both cohorts.

### 2.4 Image acquisition in PET cohort

All imaging was performed on the same PET/MR scanner (uPMR790, United Imaging Healthcare, Shanghai, China). For Aβ PET, each participant received an intravenous injection of ^18^F-AV45 (370 MBq ± 10%), followed by a 50-min resting period, and then underwent a 20-min PET acquisition on the PET/MR system. For tau PET, each participant received an intravenous injection of ^18^F-APN1607 (370 MBq ± 10%), followed by a 90-min resting period before a 20-min PET scan. Simultaneous multiparametric MR sequences were acquired using a 3.0-T field-strength scanner equipped with a 24-channel head–neck coil to improve signal-to-noise ratio. MR sequences included T1-weighted imaging (T1WI), T2-weighted imaging (T2WI), fluid-attenuated inversion recovery (FLAIR), and diffusion-weighted imaging (DWI), which were used for visual inspection of brain images to exclude participants who did not meet the eligibility criteria. For subjects visually rated as Aβ-positive, the mean value of cortical composite (Brendel et al., 2015) standardized uptake value ratios (SUVR) were further calculated using the cerebellar cortex as a reference. Due to the high heterogeneity in the cortical distribution of tau deposition across individuals, it was not feasible to apply a single standardized region of interest for the SUVR measurement. Therefore, staging of tau pathology in the MCI group was performed according to the biological staging scheme (Jack et al., 2024).

### 2.5 Statistical analysis

#### 2.5.1 Cross-cohort synthesis plan

Analyses were prespecified and applied in parallel in both cohorts. We report cohort-specific models and synthesize concordance across cohorts by direction and pattern of effects rather than by direct comparison of absolute scores or effect sizes, given differences in measurement batteries and sampling. Effect estimates are presented as odds ratios (ORs) or beta coefficients with 95% confidence intervals (CIs). Supplementary Table S4 summarizes shared elements and cohort-specific implementations, and detailed definitions appear in Sections 2.1–2.4.

#### 2.5.2 General procedures

For both the CHARLS 2018 cohort and the PET cohort, normally distributed quantitative variables were summarized as mean and standard deviation, and categorical variables as counts or percentages. Within-cohort group comparisons used the Mann–Whitney U or Wilcoxon test for continuous variables and the χ^2^ test for categorical variables. All analyses in this study were carried out using R v4.2.2. Statistical significance was set at *p*-value < 0.05. Because both datasets were pre-existing and had fixed sample sizes, no a priori sample size calculation was performed. Consistent with recommendations for observational research, we focus on effect-size estimates with 95% CIs and interpret results with attention to precision.

#### 2.5.3 Analyses in the CHARLS population-based cohort

For the analysis in the CHARLS 2018 cohort, to explore the association between CR and cognitive impairment, three binary logistic regression models were constructed using the R package “survey” v4.2.1 (10.32614/CRAN.package.survey), with low CR as the reference category. Marital status, self-reported health and self-rated memory did not differ significantly between cognitive status groups (Table 1) and, consistent with our prespecified covariate selection rule described above, were not included in the final multivariable models. Model 1 was adjusted for age and gender. Model 2 was further adjusted for self-reported hearing and self-reported eyesight based on Model 1. Model 3 was further adjusted for residence and sleeping hours based on Model 2. Adjusted ORs and 95% CI were calculated to explore the association between CR and cognitive impairment. Considering the potential confounding effects of various covariates in the CHARLS 2018 cohort, the present study further employed weighted logistic regression analysis to verify the association between CR and cognitive impairment after controlling for the covariates included in Model 3. The results were presented as a forest plot using the R package “forestplot” v3.1.3 (10.32614/CRAN.package.forestplot). After smoothing the predictive model using the locally estimated scatterplot smoothing algorithm in the R package “ggplot2” v3.4.4 (10.32614/CRAN.package.ggplot2), a predictive curve was plotted. Linear regression analysis was employed to explore the independent association between CR and the neuropsychological test scores of different cognitive domains, with low CR as the reference, using the R package “survey” v4.2.1 (10.32614/CRAN.package.survey). Three different models were constructed with covariate adjustments conducted in the same manner as described for the logistic regression models above. Taking the presence of depressive symptoms as a reference, the mediating role of depressive symptoms in the relationship between CR and cognitive impairment or domain-specific cognitive function was explored using the R package “mediation” v4.5.0 (10.32614/CRAN.package.mediation). Only the neuropsychological tests that were statistically significantly associated with CR were included in the subsequent mediation analysis. After stratifying participants into the normal cognitive group and the cognitive impairment group, the associations between CR and domain-specific cognitive performance, as well as the mediating effects of depressive symptoms, were examined separately in each group, following similar procedures described above.

**Table 1:** Demographic and clinical characteristics of cognitively impaired and cognitively normal participants in different cohorts.

**Participants from the CHARLS 2018 cohort (*****n*** = **1,636)**			
**Characteristics**	**Cognitive impairment (*****n*** = **699)**	**Cognitively normal (*****n*** = **937)**	***p-value***
Age, years (mean ± SD)	69.78 ± 5.04	69.38 ± 4.85	0.103
**Age (** ***n*** **, %)**			0.283
50–64	122 (17.5)	168 (17.9)	
65–74	433 (61.9)	605 (64.6)	
≥75	110 (20.6)	127 (17.5)	
Gender (male, %)	482 (69.0)	617 (65.8)	0.204
Education, level (mean ± SD)	5.08 ± 1.35	4.09 ± 1.51	< 0.001^***^
MMSE (mean ± SD)	22.52 ± 2.76	26.84 ± 1.98	< 0.001^***^
**Marital status (** ***n*** **, %)**			0.456
Married living with spouse	544 (77.8)	746 (79.6)	
Married not living spouse	28 (4.0)	33 (3.5)	
Separated	1 (0.1)	6 (0.6)	
Divorced	10 (1.4)	18 (1.9)	
Widowed	115 (16.5)	133 (14.2)	
Never married	1 (0.1)	1 (0.1)	
**Self-reported health status (** ***n*** **, %)**			0.15
Very good	76 (10.9)	100 (10.7)	
Good	86 (12.3)	135 (14.4)	
Fair	360 (51.5)	498 (53.1)	
Poor	131 (18.7)	166 (17.7)	
Very poor	46 (6.6)	38 (4.1)	
**Self-reported memory condition (** ***n*** **, %)**			0.909
Excellent	4 (0.6)	7 (0.7)	
Very good	35 (5.0)	55 (5.9)	
Good	65 (9.3)	90 (9.6)	
Fair	457 (65.4)	610 (65.1)	
Poor	138 (19.7)	175 (18.7)	
**Self-reported hearing (** ***n*** **, %)**			0.042^*^
Excellent	8 (1.1)	15 (1.6)	
Very good	93 (13.3)	139 (14.8)	
Good	107 (15.3)	191 (20.4)	
Fair	416 (59.5)	501 (53.5)	
Poor	75 (10.7)	91 (9.7)	
**Self-reported Eyesight (** ***n*** **, %)**			0.056
Excellent	8 (1.1)	24 (2.6)	
Very good	84 (12.0)	124 (13.2)	
Good	114 (16.3)	182 (19.4)	
Fair	397 (56.8)	499 (53.3)	
Poor	96 (13.7)	108 (11.5)	
**Sleeping hours (** ***n*** **, %)**			0.006^**^
< 6 h	234 (33.5)	307 (32.8)	
>8 h	54 (7.7)	39 (4.2)	
6–8 h	411 (58.8)	591 (63.1)	
**Residence (** ***n*** **, %)**			< 0.001^***^
City	143 (20.5)	353 (37.7)	
Semi-rural	55 (7.9)	98 (10.5)	
Rural	500 (71.5)	484 (51.7)	
Special	1 (0.1)	2 (0.2)	
Cognitive reserve (high, %)	31 (4.4)	107 (11.4)	< 0.001^***^
Depressive symptoms (present, %)	243 (34.8)	226 (24.1)	< 0.001^***^
**Participants from the PET cohort (*****n*** = **100)**			
**Characteristics**	**Mild cognitive impairment**	**Cognitively normal**	***p-value***
	**(*****n*** = **50)**	**(*****n*** = **50)**	
Age, years (mean ± SD)	68.38 ± 8.17	64.92 ± 7.37	0.028^*^
**Age (** ***n*** **, %)**			0.037^*^
50–64	13 (26.0)	25 (50.0)	
65–74	25 (50.0)	19 (38.0)	
≥75	12 (24.0)	6 (12.0)	
Gender (male, %)	30 (60.0)	27 (54.0)	0.545
Education, years (mean ± SD)	11.86 ± 3.57	13.56 ± 2.89	0.010^**^
MMSE (mean ± SD)	24.82 ± 2.61	28.56 ± 1.23	0.000^***^
MoCA (mean ± SD)	19.42 ± 3.21	24.74 ± 1.69	0.000^***^
CRI–total (mean ± SD)	103.68 ± 11.17	110.34 ± 10.86	0.003^**^
Cognitive reserve (high, %)	16(32.00)	33(66.00)	0.001^***^
Depressive symptoms (present, %)	19(38.00)	9(18.00)	0.026^*^
Amyloid PET SUVR (mean ± SD)	2.27 ± 0.46	NA	NA
**Tau PET level (** ***n*** **, %)**			
Stage A	9(18%)	NA	NA
Stage C	41(82%)	NA	NA

MMSE, Mini-Mental State Examination; MoCA, Montreal Cognitive Assessment; CRI, cognitive reserve index; PET, positron emission tomography; SUVR, standardized uptake value ratios.

#### 2.5.4 Analyses in the Clinical PET cohort

Data analysis in the PET cohort followed procedures similar to those used in the CHARLS 2018 cohort, and the same R package was applied to the corresponding analysis. To explore the association between CR and MCI or tau staging level, binary logistic regression analyses were conducted with low CR as the reference category after adjusting for age and gender. The linear regression analysis was employed to explore the independent association between CR and the neuropsychological test scores of different cognitive domains, as well as the ^18^F-AV45 PET SUVR value, using low CR as the reference after adjusting for age and gender. In this retrospective PET cohort, because MCI was defined by domain-specific neuropsychological test results rather than global cognitive function, we additionally examined the correlations of CR with MMSE and MoCA. Taking the presence of depressive symptoms as a reference, the mediating role of depressive symptoms in the relationship between CR and cognitive performance, as well as the Aβ PET SUVR value was explored. Only the neuropsychological tests that were statistically significantly associated with CR were included in the subsequent mediation analysis. The association analysis and the mediation analysis were further performed in both the Aβ-negative cognitively normal subgroup and the Aβ-positive MCI group.

## 3 Results

### 3.1 CHARLS 2018 population-based cohort

#### 3.1.1 Demographic and clinical characteristics

As shown in Table 1, all subjects were divided into two groups according to their educational level and MMSE scores in the CHARLS 2018 cohort, with 937 in the cognitively normal group and 699 in the cognitive impairment group. Age and sex were comparable between groups. Both the mean age and the distribution across age strata (50–64, 65–74, ≥75 years), as well as the sex composition, showed no significant differences. Education, residence, sleeping hours, depressive symptoms, and CR showed significant differences between cognitively normal individuals and individuals with cognitive impairment. Notably, the proportion of subjects with depressive symptoms in the cognitive impairment group was significantly higher than in the cognitively normal group.

As shown in Table 2, 519 cognitively normal participants and 397 participants with cognitive impairment were included in the domain-specific cognitive performance analysis in the CHARLS 2018 cohort. Individuals with cognitive impairment scored significantly lower than cognitively normal participants across all cognitive domains, including immediate memory, delayed memory, recognition memory, episodic memory, verbal fluency, and reasoning ability in the CHARLS study. Supplementary Table S5 summarizes the demographic and clinical characteristics of the 916 participants from the CHARLS 2018 cohort included in the cognitive domain analysis, stratified into cognitively impaired and cognitively normal groups.

**Table 2:** Neuropsychological test results in different cognitive domains: comparison between cognitively impaired and cognitively normal groups.

**Participants from the CHARLS 2018 cohort (*****n*** = **936)**			
**Neurocognitive Variables**	**Cognitive impairment**	**Cognitively normal**	***p*** **-value**
	**(*****n*** = **397)**	**(*****n*** = **519)**	
Immediate memory	15.11 ± 4.16	16.96 ± 4.42	< 0.001^***^
Delayed memory	4.65 ± 2.25	5.52 ± 2.16	< 0.001^***^
Recognition memory	18.36 ± 2.37	18.94 ± 1.67	< 0.001^***^
Episodic memory	38.11 ± 6.99	41.42 ± 6.80	< 0.001^***^
Verbal fluency	13.26 ± 4.62	14.77 ± 5.14	< 0.001^***^
Reasoning ability	9 (18.00)	19 (38.00)	0.026^*^
**Participants from the PET cohort (*****n*** = **100)**			
**Neurocognitive Variables**	**Mild cognitive impairment** **(*n* = 50)**	**Cognitively normal** **(*n* = 50)**	***p-value***
Immediate memory	12.24 ± 3.37	16.52 ± 3.18	< 0.001^***^
Delayed memory	1.48 ± 1.83	5.62 ± 1.28	< 0.001^***^
Recognition memory	16.86 ± 3.39	21.22 ± 1.68	< 0.001^***^
z_Episodic memory	−0.71 ± 0.81	0.71 ± 0.57	< 0.001^***^
TMT A (time, s)	66.18 ± 24.84	47.50 ± 13.57	< 0.001^***^
TMT B (time, s)	134.18 ± 77.70	90.40 ± 36.73	< 0.001^***^
z_Executive function	−0.38 ± 1.19	0.38 ± 0.56	< 0.001^***^
BNT	20.50 ± 3.52	23.26 ± 2.97	< 0.001^***^
Verbal fluency	15.62 ± 4.89	18.10 ± 3.99	0.007^**^
z_Language function	−0.40 ± 0.91	0.40 ± 0.94	< 0.001^***^
DSF	7.26 ± 1.47	7.60 ± 1.25	0.215
DSB	4.20 ± 1.11	4.98 ± 1.39	0.003^**^
SDMT	28.30 ± 10.93	37.90 ± 9.60	< 0.001^***^
z_Attention	−0.43 ± 0.93	0.43 ± 0.88	< 0.001^***^
CDR–SB	1.81 ± 1.01	NA	NA

^*^p < 0.05, ^**^p < 0.01, ^***^p < 0.001.

TMT, trail making test; BNT, Boston naming test; AFT, animal fluency test; DSF, digit span forward; DSB, digit span backward; SDMT, symbol digit modalities test; CDR-SB, clinical dementia rating scale sum of boxes.

#### 3.1.2 Association between CR and global cognition

**Figure 1:**
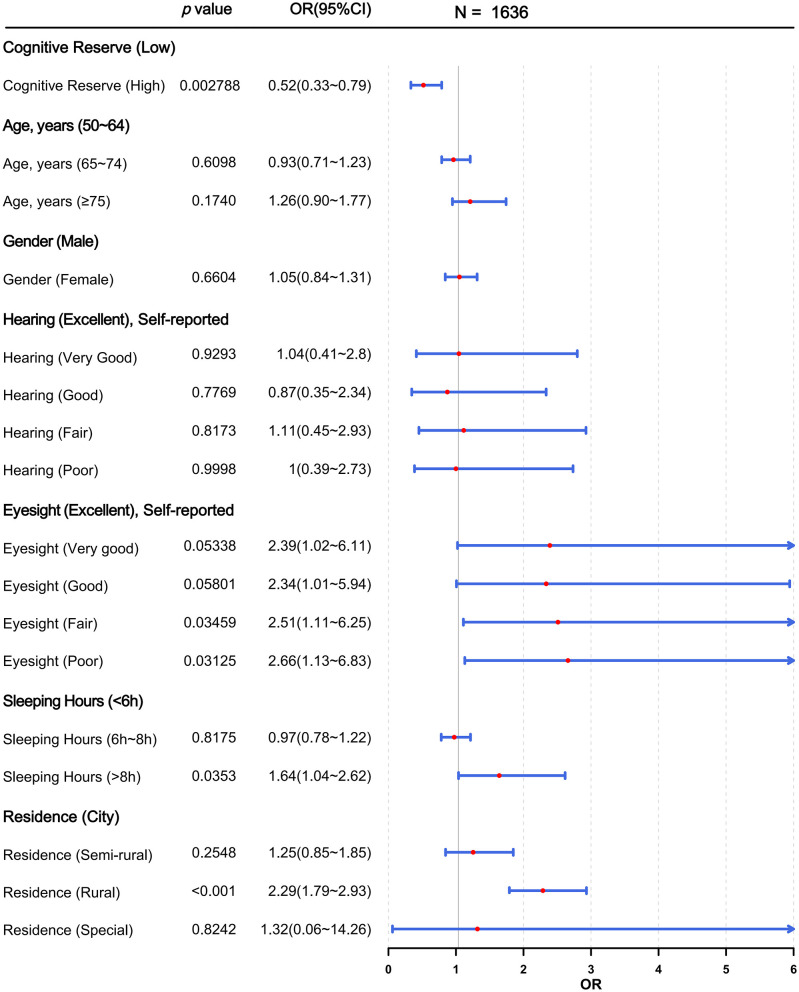
Forest plot displaying adjusted odds ratios (ORs) and 95% confidence intervals (CIs) for predictors of cognitive impairment after adjustment of age, gender, self-reported hearing, self-reported eyesight, residence, and sleeping hours (*n* = 1,636).

**Figure 2:**
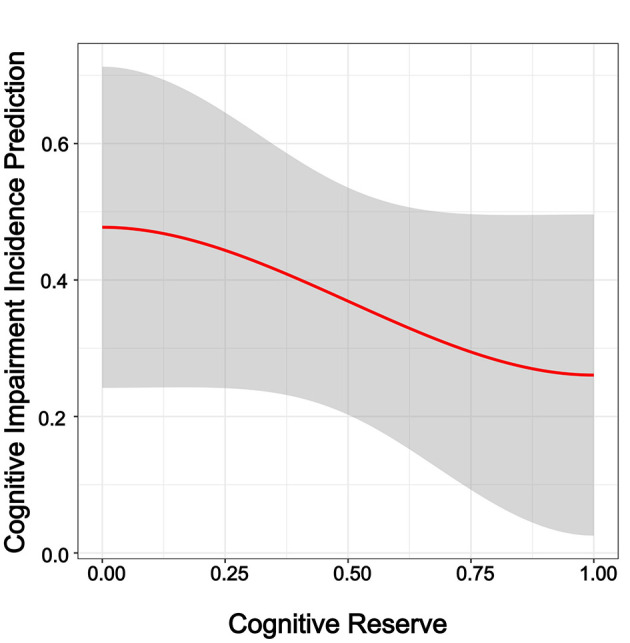
Prediction curve illustrating the probability of cognitive impairment according to cognitive reserve and depressive symptoms, after smoothing by the locally estimated scatterplot smoothing algorithm.

Three binary logistic regression models were built to explore the correlation between CR and cognitive impairment in the CHARLS 2018 cohort. As shown in Table 3, Model 1 (OR = 0.36, 95% CI = 0.23–0.54, *p* < 0.0001), Model 2 (OR = 0.38, 95% CI = 0.25–0.57, *p* < 0.0001), and Model 3 (OR = 0.52, 95% CI = 0.33–0.79, *p* < 0.01) all demonstrated significant associations. The results indicate that CR was significantly associated with cognitive impairment and served as a protective factor. To further demonstrate the effect of CR on cognitive impairment after adjusting for covariates in Model 3, weighted logistic regression analysis showed that high CR remained strongly associated with a lower likelihood of cognitive impairment, confirming its buffering function (Figure 1; *p* < 0.01, OR < 1). In addition, suboptimal vision (fair or poor eyesight), excessive sleep duration (>8 h), and rural residence were identified as risk factors for cognitive impairment (Figure 1; *p* < 0.05, OR > 1). The predictive curve showed that as CR improved, the probability of cognitive impairment decreased (Figure 2).

**Table 3:** Associations of cognitive reserve with cognitive performance in the CHARLS 2018 cohort.

**Neurocognitive Variables**	**Model 1**	**Model 2**	**Model 3**
	β**/OR [95% CI]**	β**/OR [95% CI]**	β**/OR [95% CI]**
**The overall included participants (*****n*** = **1,636)**			
Cognitive impairment	0.36^***^ [0.23, 0.54]	0.38^***^ [0.25, 0.57]	0.52^**^ [0.33, 0.79]
**Participants in the domain-specific cognitive performance**			
**analysis (*****n*** = **936)**			
Immediate memory	2.13^***^ [1.08, 3.18]	1.99^***^ [0.94, 3.04]	1.53^**^ [0.46, 2.60]
Delayed memory	0.96^**^ [0.43, 1.50]	0.86^**^ [0.33, 1.40]	0.58^*^ [0.04, 1.12]
Recognition memory	0.72^**^ [0.24, 1.21]	0.63^*^ [0.15, 1.12]	0.50 [−0.04, 1.00]
Episodic memory	3.82^***^ [2.14, 5.49]	3.49^***^ [1.81, 5.16]	2.60^**^ [0.89, 4.30]
Verbal fluency	3.25^***^ [2.08, 4.43]	3.14^***^ [1.96, 4.32]	2.26^***^ [1.07, 3.45]
Reasoning ability	0.88^***^ [0.61, 1.15]	0.87^***^ [0.60, 1.14]	0.62^***^ [0.35, 0.89]
**Cognitively normal group (*****n*** = **519)**			
Immediate memory	1.44^*^ [0.25, 2.62]	1.20^*^ [0.01, 2.38]	0.96 [−0. 26, 2.17]
Delayed memory	0.72^*^ [0.14, 1.30]	0.64^*^ [0.06, 1.22]	0.42 [−0.18, 1.01]
Recognition memory	0.51^*^ [0.06, 0.96]	0.44^*^ [−0.01, 0.89]	0.37 [−0.10, 0.83]
Episodic memory	2.66^**^ [0.84, 4.48]	2.27^*^ [0.46, 4.09]	1.74 [−0.12, 3.60]
Verbal fluency	2.35^***^ [0.97, 3.73]	2.20^***^ [0.82, 3.58]	1.47^*^ [0.073, 2.86]
Reasoning ability	0.68^***^ [0.36, 0.99]	0.68^***^ [0.37, 0.99]	0.50^***^ [0.18, 0.812]
**Cognitive impairment group (*****n*** = **397)**			
Immediate memory	2.45^*^ [0.15, 4.75]	2.62^*^ [0.24, 5.00]	2.37 [−0.03, 4.76]
Delayed memory	0.76 [−0.49, 2.01]	0.57 [−0.70, 1.85]	0.45 [−0.83, 1.74]
Recognition memory	0.835 [−0.48, 2.15]	0.55 [−0.81, 1.91]	0.46 [−0.92, 1.83]
Episodic memory	4.04^***^ [0.18, 7.90]	3.75 [−0.23, 7.73]	3.28 [−0.71, 7.27]
Verbal fluency	5.30^***^ [2.79, 7.82]	4.74^***^ [2.15, 7.33]	4.34^***^ [1.76, 6.93]
Reasoning ability	1.11^***^ [0.54, 1.68]	1.07^***^ [0.485, 1.66]	0.88^**^ [0.30, 1.45]

^*^p < 0.05, ^**^p < 0.01, ^***^p < 0.001. Model 1 was adjusted by age and gender. Model 2 was adjusted by adding self-reported hearing and self-reported eyesight based on Model 1. Model 3 further adjusted the residence and sleeping hours based on Model 2.

OR, odds ratio; CI, confidence interval.

#### 3.1.3 Relationship between CR and domain-specific cognitive performance

In the CHARLS 2018 cohort, CR was significantly positively associated with scores for immediate and delayed memory in all three models, but was related to phrase recognition only in Model 1 and Model 2 (Table 3). Furthermore, CR was significantly positively associated with episodic memory, verbal fluency, and reasoning ability in all three models (Table 3). CR showed a significant positive correlation with verbal fluency and reasoning ability in all three models in both the cognitive impairment group and the cognitively normal group (Table 3). After adjusting for age, sex, self-reported hearing, and self-reported eyesight in Model 2, CR was positively associated with immediate memory in both the cognitively normal group and the cognitive impairment group (Table 3).

#### 3.1.4 Mediation effects of depressive symptoms between CR and cognitive performance

As shown in Table 4, when the presence of depressive symptoms was used as the reference, the absence of depressive symptoms mediated the relationship between CR and cognitive impairment among 1,636 subjects in the CHARLS 2018 cohort, and the indirect effect of CR on cognitive impairment through depressive symptoms accounted for 7.2% of the total effect. Among the 916 subjects included in the analysis of cognitive function across different cognitive domains in the CHARLS 2018 cohort, when the presence of depressive symptoms was used as the reference, the absence of depressive symptoms was found to mediate the association between CR and immediate memory, episodic memory, verbal fluency, and reasoning ability, with the indirect effects accounting for 6.2%, 5.1%, 3.7%, and 3.1% of the total effects, respectively (Table 4). However, the mediating effects of depressive symptoms between CR and different cognitive domains were not observed in either the cognitive impairment group or the cognitively normal group (Supplementary Table S6).

**Table 4:** Mediation effects of depressive symptoms on the association between cognitive reserve and cognitive performance in the overall CHARLS 2018 cohort.

**Neurocognitive Variables**	**ACME**		**ADE**		**Total Effect**		**Prop. Mediated**	
	**Estimate value**	**[95% CI]**	**Estimate value**	**[95% CI]**	**Estimate value**	**[95% CI]**	**Estimate value**	**[95% CI]**
Cognitive impairment	0.016^**^	[0.006, 0.028]	0.205^**^	[0.130, 0.279]	0.222^**^	[0.146, 0.296]	0.072^**^	[0.028, 0.141]
Immediate memory	0.139^*^	[0.010, 0.298]	1.999^**^	[1.002, 2.987]	2.138^**^	[1.117, 3.132]	0.062^*^	[0.004, 0.166]
Delayed memory	0.050	[−0.001, 0.117]	0.915^**^	[0.418, 1.40]	0.965^**^	[0.467, 1.455]	0.049	[−0.001, 0.152]
Recognition memory	0.017	[−0.017, 0.063]	0.701^**^	[0.420, 0.966]	0.718^**^	[0.442, 0.984]	0.020	[−0.024, 0.096]
Episodic memory	0.206^*^	[0.014, 0.449]	3.689^**^	[2.108, 5.238]	3.821^**^	[2.283, 5.344]	0.051^**^	[0.003, 0.135]
Verbal fluency	0.127^*^	[0.003, 0.284]	3.115^**^	[1.627, 4.595]	3.242^**^	[1.733, 4.736]	0.037^*^	[0.001, 0.105]
Reasoning ability	0.029^*^	[0.000, 0.065]	0.855^**^	[0.567, 1.145]	0.884^**^	[0.587, 1.177]	0.031^*^	[0.000, 0.080]

^*^p < 0.05, ^**^p < 0.01.

N = 1,636 in the analysis of cognitive impairment. N = 916 in the analysis of domain-specific cognitive performance.

ACME, average causal mediation effects (indirect effect). ADE, average direct effects. Prop. Mediated, proportion mediated. CI, confidence interval.

### 3.2 Clinical PET cohort

#### 3.2.1 Demographic and clinical characteristics

Among the 100 participants from the PET cohort (Table 1), those with MCI (*n* = 50) were older and had fewer years of education compared with cognitively normal individuals (*n* = 50), while sex distribution was comparable. Compared with the CHARLS 2018 cohort, participants in the clinical PET cohort were younger and had markedly higher educational levels. According to the educational level shown in Supplementary Table S2, most participants in the CHARLS 2018 cohort had only elementary or middle school education, while all participants in the PET cohort had education at or above the middle school level.

The MCI group also showed significantly lower CRI-total scores and a smaller proportion with high CR compared with cognitively normal individuals (Table 1). Depressive symptoms were more prevalent in MCI than in cognitively normal participants (Table 1). MCI patients showed poorer memory performance, executive function, language function, and attention compared with cognitively normal participants, based on various neuropsychological test results across different cognitive domains (Table 2).

**Figure 3:**
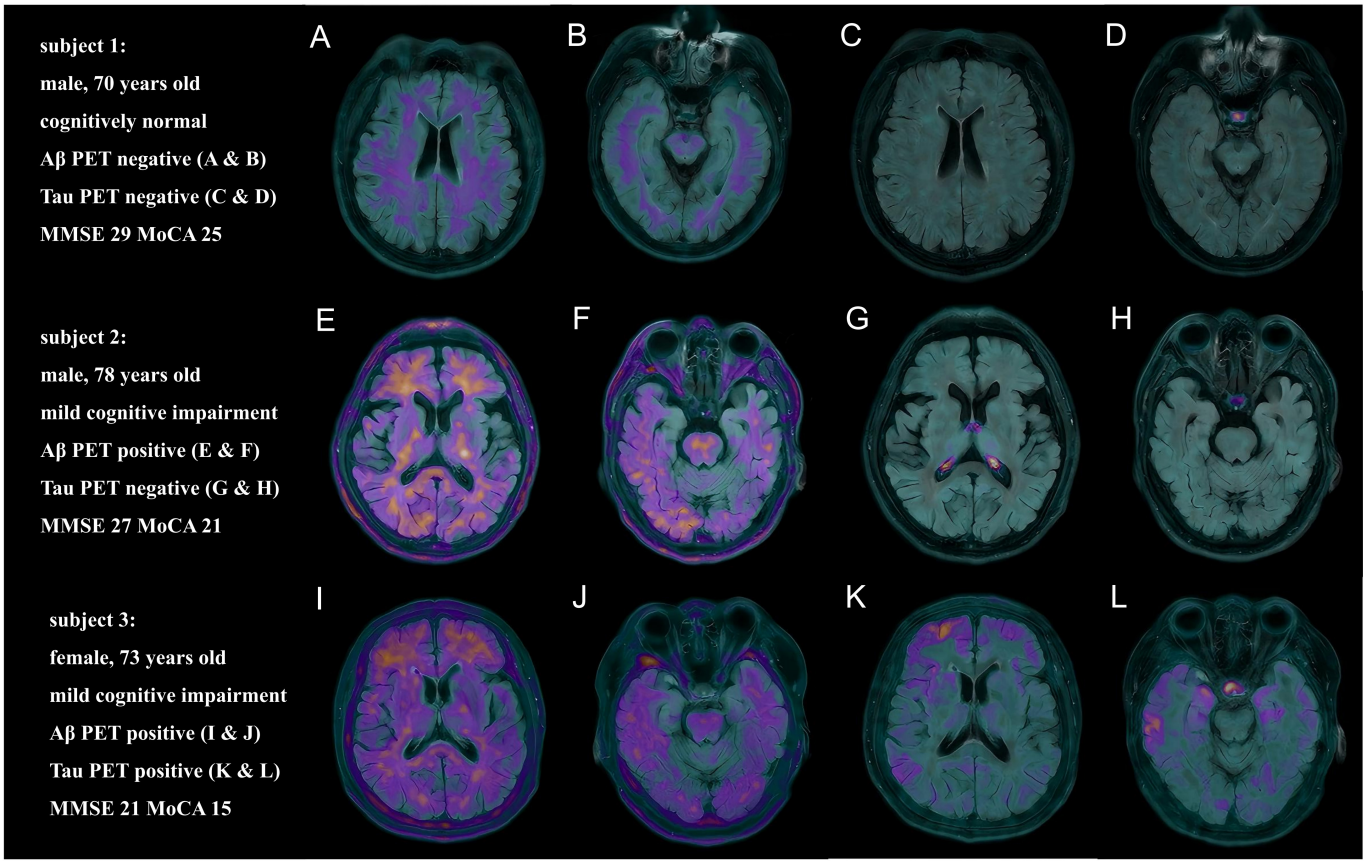
Representative ^18^F-AV45 PET and ^18^F-APN1607 PET uptake modalities for three participants in the PET cohort are shown. Subject 1: a cognitively normal male participant with no Aβ deposition on PET **(A, B)** and no tau deposition on PET **(C, D)**; Subject 2: a male patient with mild cognitive impairment (MCI), with significant Aβ deposition (SUVR mean = 2.22) on PET **(E, F)** but no tau deposition **(G, H)**, indicating stage A in the biological staging scheme for tau PET; Subject 3: an MCI female patient with significant Aβ deposition (SUVR mean = 2.01) on PET **(I, J)**; moderate tau deposition (SUVR max = 1.88) was observed in both the medial temporal lobe and neocortical regions on PET, indicating stage C in the biological staging scheme for tau PET **(K, L)**. Each panel reports age, sex, education, global cognitive status (cognitively normal or MCI), and scores on the Mini-Mental State Examination (MMSE) and the Montreal Cognitive Assessment (MoCA). Aβ, amyloid beta; PET, positron emission tomography; SUVR, standard uptake value ratio.

The mean value of Aβ PET SUVR was 2.27 ± 0.46 in the MCI group. Furthermore, most MCI patients were categorized at stage C (82%), with the rest of the participants classified into stage A (18%) according to tau PET (Jack et al., 2024). PET images and corresponding clinical information of three participants, each with different amyloid and tau statuses and who underwent both ^18^F-AV45 and ^18^F-APN1607 PET, are presented in Figure 3.

#### 3.2.2 Association between CR and global cognition

As shown in Table 5, higher CR was strongly associated with better cognitive outcomes in participants from the PET cohort. Specifically, increased CR was linked to a reduced likelihood of MCI (OR = 0.230, 95% CI = 0.097–0.547, *p* < 0.001). In addition, CR was positively associated with global cognitive test scores, as reflected by higher MMSE (β = 2.911, 95% CI = 2.045–3.776, *p* < 0.001) and MoCA scores (β = 3.651, 95% CI = 2.444–4.859, *p* < 0.001).

**Table 5:** Associations of cognitive reserve with cognitive performance in the PET cohort.

**Clinical Variables**	**Overall participants (*n* = 100)**	**Cognitively normal** **(*n* = 50)**	**MCI (*n* = 50)**
	β**/OR [95% CI]**	β**/OR [95% CI]**	β**/OR [95% CI]**
MCI	0.230^***^ [0.097, 0.547]	NA	NA
MMSE	2.911^***^ [2.045, 3.776]	1.067^**^ [0.425, 1.709]	2.816^***^ [1.576, 4.055]
MoCA	3.651^***^ [2.444, 4.859]	1.083^*^ [0.111, 2.056]	3.352^***^[1.780, 4.925]
Immediate memory	2.581^***^ [1.215, 3.947]	1.708 [−0.006, 3.422]	1.156 [−0.913, 3.226]
Delayed memory	1.389^**^ [0.427, 2.351]	0.112 [−0.669, 0.894]	0.036 [−1.076, 1.148]
Recognition memory	1.713^*^ [0.398, 3.027]	0.137 [−0.900, 1.175]	0.549 [−1.583, 2.680]
z_Episodic memory	0.635^**^ [0.274, 0.995]	0.219 [−0.111, 0.548]	0.194 [−0.308, 0.697]
TMT A	−16.752^***^ [−24.281, −9.222]	−5.348 [−13.354, 2.658]	−22.353^**^ [−35.485, −9.221]
TMT B	−50.566^***^ [−73.597, −27.534]	−35.347^**^ [−54.922, −15.772]	−53.294^*^ [−99.025, −7.563]
z_Executive function	0.825^***^ [0.477, 1.174]	0.499^**^ [0.194, 0.804]	0.927^**^ [0.251, 1.603]
BNT	2.970^***^ [1.695, 4.245]	3.012^***^ [1.399, 4.625]	1.714 [−0.414, 3.842]
verbal fluency	3.747^***^ [2.076, 5.419]	3.235^**^ [0.995, 5.476]	3.491^*^ [0.619, 6.364]
z_Language function	1.020^***^ [0.682, 1.358]	0.949^***^ [0.444, 1.453]	0.790^**^ [0.290, 1.291]
DSF	0.976^***^ [0.480, 1.473]	0.949^**^ [0.297, 1.600]	1.000^*^ [0.148, 1.852]
DSB	0.550^*^ [0.042, 1.058]	0.639 [−0.161, 1.438]	−0.023 [−0.728, 0.681]
SDMT	11.023^***^ [7.391, 14.655]	8.878^***^ [4.389, 13.367]	9.356^**^ [3.072, 15.639]
z_Attention	1.018^***^ [0.702, 1.333]	0.849^***^ [0.447, 1.250]	0.838^**^ [0.306, 1.369]
CDR-SB	NA	NA	−0.827^**^ [−1.377, −0.276]
Amyloid PET SUVR	NA	NA	0.026 [−0.263, 0.314]
Tau PET level	NA	NA	0.941 [0.189, 4.689]

^*^p < 0.05, ^**^p < 0.01, ^***^p < 0.001, after the adjustment of age and gender.

MCI, mild cognitive impairment; MMSE, Mini-Mental State Examination; MoCA, Montreal Cognitive Assessment; TMT, trail making test; BNT, Boston naming test; DSF, digit span forward; DSB, digit span backward; SDMT, symbol digit modalities test; CDR-SB, clinical dementia rating scale sum of boxes; PET, positron emission tomography; SUVR, standardized uptake value ratios; OR, odds ratio; CI, confidence interval; NA, not applicable.

#### 3.2.3 Relationship between CR and domain-specific cognitive performance

As shown in Table 5, higher CR was significantly associated with better cognitive performance across multiple cognitive domains in the PET cohort. CR was positively related to all subset neuropsychological test results and to the z-scores of all cognitive domains in the overall sample. In subgroup analyses, CR was not significantly related to any memory indices or to the Digit Span Backward (DSB) test in either cognitively normal participants or those with MCI. CR remained positively associated with the z-scores of executive function, language function, and attention in both subgroups. No associations were observed between CR and amyloid SUVR or tau levels.

#### 3.2.4 Mediation effects of depressive symptoms between CR and cognitive performance

The mediation analysis results for overall participants from the PET cohort are presented in Table 6. No significant mediation was observed for the association between CR and MCI status when depressive symptoms were examined as the mediator. However, significant indirect effects were identified for global cognitive rating scales (MMSE and MoCA), with depressive symptoms accounting for 23.1% to 30.8% of the total effect. Recognition memory showed full mediation, with 65.4% of the effect explained through depressive symptoms. Evidence of partial mediation was also found for episodic memory (z-score), TMT-A, TMT-B, executive function (z-score), and Digit Span Forward (DSF), with proportions mediated ranging from 27.7% to 37.6%. In contrast, no significant mediation was observed for immediate memory, delayed memory, language-related cognitive tests, DSB, SDMT, or attention (z-score).

**Table 6:** Mediation effects of depressive symptoms on the association between cognitive reserve and cognitive performance in the overall PET cohort (*n* = 100).

**Neurocognitive Variables**	**ACME**		**ADE**		**Total Effect**		**Prop. Mediated**	
	**Estimate value**	**[95% CI]**	**Estimate value**	**[95% CI]**	**Estimate value**	**[95% CI]**	**Estimate value**	**[95% CI]**
MCI	−0.033	[−0.143, 0.051]	−0.283^**^	[−0.490, −0.072]	−0.316^***^	[−0.499, −0.148]	0.093	[−0.194, 0.595]
MMSE	0.692^**^	[0.219,1.365]	2.242^***^	[1.308, 3.258]	2.933^***^	[2.101, 3.957]	0.231^**^	[0.055, 0.453]
MoCA	1.144^**^	[0.453, 2.092]	2.527^***^	[1.263, 3.894]	3.672^***^	[2.406, 4.893]	0.308^**^	[0.124, 0.568]
Immediate memory	0.649	[−0.016, 1.589]	1.972^**^	[0.472, 3.598]	2.622^**^	[1.236, 3.990]	0.244	[−0.009, 0.699]
Delayed memory	0.392	[−0.076, 1.027]	1.025	[−0.030, 2.162]	1.412^**^	[0.442, 2.383]	0.274	[−0.059, 1.013]
Recognition memory	1.131^**^	[0.420, 2.131]	0.580	[−0.765, 2.037]	1.711^*^	[0.392, 3.018]	0.654^*^	[0.205, 2.265]
z_Episodic memory	0.243^**^	[0.059, 0.517]	0.400^*^	[0.011, 0.821]	0.643^**^	[0.271, 1.003]	0.372^**^	[0.083, 0.961]
TMT A	−5.028^*^	[−10.176, −1.041]	−12.229^**^	[−20.617, −3.197]	−17.257^***^	[−25.330, −9.423]	0.277^*^	[0.061, 0.716]
TMT B	−17.582^**^	[−33.199, −5.281]	−33.746^**^	[−57.790, −7.824]	−51.328^***^	[−75.022, −28.539]	0.328^**^	[0.105, 0.077]
z_Executive function	0.272^**^	[0.086, 0.544]	0.559^**^	[0.189, 0.964]	0.831^***^	[0.473, 1.197]	0.322^**^	[0.100, 0.675]
BNT	0.415	[−0.187, 1.193]	2.550^***^	[1.204, 4.002]	2.965^***^	[1.739,4.196]	0.137	[−0.064, 0.434]
Verbal fluency	0.174	[−0.660, 1.121]	3.593^***^	[1.804, 5.526]	3.767^***^	[2.150,5.417]	0.434	[−0.189, 0.331]
z_Language function	0.594	[−0.486, 1.934]	6.148^***^	[3.763, 8.726]	6.742^***^	[4.617,8.922]	0.086	[−0.074, 0.307]
DSF	0.373^**^	[0.113, 0.749]	0.606^*^	[0.089, 0.163]	0.979^***^	[0.479, 1.482]	0.376^**^	[0.111, 0.866]
DSB	0.158	[−0.082, 0.471]	0.393	[−0.146, 0.973]	0.551^*^	[0.061, 1.044]	0.279	[−0.236, 1.694]
SDMT	1.071	[−0.760, 3.378]	10.104^***^	[6.026, 14.505]	11.174^***^	[7.526, 14.893]	0.094	[−0.071, 0.328]
z_Attention	0.130	[−0.027, 0.343]	0.901^***^	[0.546, 1.288]	1.031^***^	[0.706, 1.351]	0.123	[−0.027, 0.340]

^*^p < 0.05, ^**^p < 0.01, ^***^p < 0.001.

ACME, average causal mediation effects (indirect effect). ADE, average direct effects. Prop. Mediated, proportion mediated; PET, positron emission tomography; MCI, mild cognitive impairment; MMSE, Mini-Mental State Examination; MoCA, Montreal Cognitive Assessment; TMT, trail making test; BNT, Boston naming test; DSF, digit span forward; DSB, digit span backward; SDMT, symbol digit modalities test; CI, confidence interval.

In subgroup analyses from the PET cohort (Table 7), no significant mediation was observed among cognitively normal Aβ PET-negative participants. In the MCI and Aβ PET-positive subgroup, mediation was generally absent, except for DSF, which showed full mediation, with 59.6% of the effect explained by depressive symptoms. Other outcomes, including TMT-B and executive function (z-score), also demonstrated significant indirect effects, although the mediated proportions (43.9% and 41.4%) did not reach statistical significance.

**Table 7:** Mediation effects of depressive symptoms on the association between cognitive reserve and cognitive performance across the PET cohort subgroups.

**Neurocognitive Variables**	**ACME**		**ADE**		**Total Effect**		**Prop. Mediated**	
	**Estimate value**	**[95% CI]**	**Estimate value**	**[95% CI]**	**Estimate value**	**[95% CI]**	**Estimate value**	**[95% CI]**
**Amyloid-negative cognitively normal group (*****n*** = **50)**								
TMT B	−6.721	[−19.103, 1.267]	−28.844^**^	[−48.367, −8.172]	−35.565^***^	[−55.191, −16.066]	0.170	[−0.037, 0.624]
z_Executive function	0.104	[−0.020, 0.292]	0.392^*^	[0.086, 0.725]	0.496^***^	[0.194, 0.803]	0.196	[−0.039, 0.670]
BNT	0.482	[−0.170, 1.420]	2.451^**^	[0.829, 4.178]	2.932^**^	[1.334,4.530]	0.149	[−0.055, 0.521]
Verbal fluency	−0.027	[−1.154, 1.026]	3.277^**^	[0.976, 5.717]	3.251^**^	[1.081,5.479]	−0.434	[−0.489, 0.382]
z_Language function	0.069	[−0.148, 0.336]	0.871^**^	[0.358, 1.417]	0.940^**^	[0.449, 1.443]	0.064	[−0.169, 0.395]
DSF	0.089	[−0.220, 0.453]	0.897^*^	[0.176, 1.669]	0.987^**^	[0.302, 1.695]	0.079	[−0.283, 0.592]
SDMT	1.235	[−0.883, 4.097]	7.980^**^	[2.804, 13.464]	9.216^**^	[4.151, 14.281]	0.122	[−0.093, 0.498]
z_Attention	0.106	[−0.088, 0.369]	0.776^**^	[0.304, 1.282]	0.882^**^	[0.421, 1.342]	0.109	[−0.097, 0.457]
**Amyloid-positive mild cognitive impairment group (*****n*** = **50)**								
TMT A	−5.553	[−13.770, 0.772]	−13.439	[−27.699, 1.904]	−18.991^**^	[−32.876, −5.264]	0.272	[−0.045, 1.207]
TMT B	−22.187^*^	[−49.365, −0.997]	−24.243	[−69.338, 24.263]	−46.430^*^	[−90.804, −2.467]	0.439	[−0.098, 2.615]
z_Executive function	0.332^*^	[0.017, 0.742]	0.455	[−0.224, 1.194]	0.788^*^	[0.107, 1.496]	0.414	[−0.028, 1.802]
Verbal fluency	0.185	[−1.078, 1.594]	3.114^*^	[0.232, 6.219]	3.299^*^	[0.638, 6.082]	0.053	[−0.472, 0.729]
z_Language function	0.059	[−0.155, 0.301]	0.683^**^	[0.177, 1.234]	0.742^**^	[0.273, 1.236]	0.076	[−0.255, 0.518]
DSF	0.552^*^	[0.100, 0.453]	0.368	[−0.407, 1.208]	0.920^*^	[0.054, 1.769]	0.596^*^	[0.093, 2.365]
SDMT	0.370	[−0.2.436, 3.464]	8.390^**^	[2.033, 15.234]	8.761^**^	[2.894, 14.898]	0.038	[−0.359, 0.493]
z_Attention	0.097	[−0.128, 0.372]	0.685^*^	[0.151, 1.261]	0.782^**^	[0.284, 1.303]	0.122	[−0.176, 0.596]
CDR-SB	−0.235	[−0.559, 0.015]	−0.511	[−1.060, −0.081]	−0.746^**^	[−1.284, −0.213]	0.297	[−0.019, 1.215]

^*^p < 0.05, ^**^p < 0.01, ^***^p < 0.001.

ACME, average causal mediation effects (indirect effect). ADE, average direct effects. Prop. Mediated, proportion mediated; PET, positron emission tomography; MCI, mild cognitive impairment; MMSE, Mini-Mental State Examination; MoCA, Montreal Cognitive Assessment; TMT, trail making test; BNT, Boston naming test; DSF, digit span forward; DSB, digit span backward; SDMT, symbol digit modalities test; CI, confidence interval.

## 4 Discussion

This study examined whether cognitive reserve (CR) is related to global and domain specific cognition in Chinese adults aged 50 to 80 years and whether depressive symptoms mediate this relationship. Using a harmonized analytic plan, we conducted parallel cohort specific analyses in the CHARLS population cohort and an independent clinical PET cohort and obtained broadly concordant results, with the PET cohort providing additional insights informed by AD biomarkers.

In the overall CHARLS 2018 sample, we found that higher CR was associated with lower odds of cognitive impairment and with better performance in memory, language, and executive function (reasoning ability). Depressive symptoms partially mediated the relationship between CR and global cognition, as well as the associations with immediate memory, episodic memory, verbal fluency, and reasoning ability. In pre-specified subgroup analyses stratified by cognitive status, CR remained positively associated with language and reasoning ability in both cognitively normal and cognitively impaired participants. However, under strict covariate adjustment, CR was not related to any memory outcomes and no mediating effects of depressive symptoms were observed in either subgroup.

In the clinical PET cohort, higher CR was associated with a lower likelihood of MCI and with higher global cognition. In the overall sample, higher CR was also related to better memory, executive, language, and attentional performance. Depressive symptoms mediated the association between CR and global cognition and showed full mediation for recognition memory. Partial mediation was observed for episodic memory, executive function, and attention. CR was not associated with Aβ burden or tau levels. In subgroup analyses stratified by amyloid status and cognitive status, CR remained positively associated with executive function, language, and attention in both the amyloid negative cognitively normal subgroup and the amyloid positive MCI subgroup, while memory measures were not related to CR in either subgroup. Mediation by depressive symptoms was observed only in the AD-related MCI subgroup and was absent in the amyloid negative cognitively normal subgroup.

Across cohorts, higher CR was associated with better cognitive performance and a lower likelihood of cognitive impairment, and in both overall samples depressive symptoms mediated these associations, predominantly partially. Stratified analyses showed a shared pattern in both cohorts, with higher CR related to better executive function and to better language, but not to memory measures. The main difference concerned mediation. No mediation was evident in stratified analyses in CHARLS, whereas mediation was observed only in the AD-related MCI subgroup in the PET cohort. Attention was assessed only in the PET cohort, so correspondence could not be evaluated for this domain.

The convergent pattern observed across both the CHARLS 2018 cohort and the clinical PET cohort, in which higher CR is associated with better performance across several cognitive domains, is in line with findings from other longitudinal and cross sectional cohorts. In another European cohort (Khalaila et al., 2024), individuals in the middle and lowest CR tertiles exhibited poorer performance in immediate memory and verbal fluency compared with those in the highest tertile. Similarly, higher CR was associated with better performance in episodic memory and executive function in a large American male cohort (Schwarz et al., 2024). Together, these converging results suggest that CR confers a general advantage across multiple cognitive domains. This behavioral pattern is consistent with current models of CR as a buffering mechanism against both normative brain aging and pathological cerebral changes, including neurodegenerative and vascular processes (Pappalettera et al., 2024; Stern et al., 2020). The efficiency-capacity account proposes that individuals with higher CR can perform routine tasks with lower neural activation and can recruit additional networks when task demands increase (Stern et al., 2023). In other words, high CR supports more economical use of brain resources in easy conditions and greater flexibility to mobilize extra neural capacity in challenging conditions (Stern, 2009). Such a mechanism provides a plausible neural explanation for why, across different populations and measurement batteries, higher CR repeatedly emerges as a correlate of better cognitive performance, as we also observed in the present study.

In both the CHARLS 2018 cohort and the clinical PET cohort, more detailed stratified analyses showed that the associations between CR and memory performance were not robust, and they did not remain after covariate adjustment within clinically defined subgroups. Only a few studies have reported that, among cognitively normal individuals, higher CR is significantly associated with better memory performance (Boyle et al., 2021; Narbutas et al., 2021). Overall, the available data suggest that the link between CR and memory, particularly in individuals with cognitive impairment, is not yet firmly established. Further investigation of the association between CR and memory function in populations with and without cognitive impairment is therefore needed in large cohorts worldwide.

The relationship between CR and depression appears bidirectional in other large population-based studies. Higher CR is associated with fewer depressive symptoms and better mental health (Porricelli et al., 2024; Zijlmans et al., 2023), and CR can attenuate the effect of cerebrovascular or degenerative changes on mood, consistent with more efficient or compensatory neural recruitment (Lin et al., 2020). At the same time, a higher depressive symptom burden predicts subsequent reductions in leisure, social and other reserve-building activities, and these activity losses in turn contribute to later cognitive decline in older adults (Wang, 2024; Hung et al., 2023; Sharifian et al., 2020), plausibly through anhedonia, fatigue and negative attentional bias that limit engagement with cognitively stimulating environments (Pizzagalli, 2014; Peckham et al., 2010). Our results across both cohorts fit within this framework by showing that depressive symptoms partially mediated the association between composite CR and cognitive performance in the overall samples. This is also consistent with previous Chinese data, as Jia et al. (2022) observed that low CR and the presence of depression had a combined effect on increasing the risk of dementia among older adults aged 65 years and above. Lara et al. (2022) also reported a statistically significant negative interaction between depression and CR on both immediate and delayed recall, indicating that at higher levels of CR, the presence of depression was associated with lower memory scores, which attenuated the memory advantage typically conferred by higher CR.

This pattern, in which depressive symptoms mediated the association between CR and cognition only in the AD-related MCI subgroup but not in the stratified analyses in CHARLS or in the Aβ PET negative cognitively normal subgroup, is consistent with emerging work on how CR interacts with specific neuropathological burdens. In the CHARLS cohort, the absence of mediation in stratified analyses may partly reflect the lack of biomarker confirmation of underlying pathology, so both cognitively impaired and cognitively normal participants may include mixtures of AD-related and non-AD-related etiologies, which could introduce heterogeneity and weaken subgroup specific effects. A follow up study in which participants underwent postmortem autopsies concluded that higher CR was associated with a lower burden of global AD pathology (Bennett et al., 2012), but showed no association with vascular disease pathology, Lewy body pathology, or hippocampal sclerosis (Li et al., 2021). Another large scale cohort study suggested that the mitigating effect of CR in slowing cognitive decline was evident only in individuals who were positive for AD pathology, while CR may not confer protection in those without such pathology (McKenzie et al., 2020). Alongside CR, the related construct of brain reserve (BR) has been proposed as a structural form of protection against cognitive decline (Groot et al., 2018). BR can be indexed by markers of structural brain integrity, including total brain volume, hippocampal volume, and WMH volume (Stern et al., 2020). In the absence of evident AD pathology, BR serves as the primary protector against neural damage (Stern et al., 2020; Groot et al., 2018). Neuroimaging evidence indicates that structural alterations, such as gray matter atrophy and WMH, are significantly associated with late-life depression (LLD) in non-dementia contexts (Invernizzi et al., 2021). As a whole, these observations suggest that, when AD pathology is absent or heterogeneous, structural BR rather than CR may play the dominant role in buffering depression related insults. This framework is compatible with the lack of robust mediation effects in the CHARLS subgroups and in the Aβ PET negative cognitively normal subgroup.

In the PET confirmed AD-related MCI group, depressive symptoms fully mediated the effect of CR on executive function and attention, suggesting that in these patients the protective influence of CR on cognition operates largely through the absence or lower burden of depressive symptoms. Depression may contribute to AD risk through chronic stress, inflammation and vascular vulnerability, but it can also emerge as a clinical manifestation of AD pathology itself (Huang et al., 2024). Shared mechanisms such as genetic susceptibility, hippocampal atrophy, network dysfunction, and hypothalamic–pituitary–adrenal axis alterations point to a complex, bidirectional relationship between depression and AD (Huang et al., 2024). Clinically, in patients along the AD continuum, depressive symptoms may precede measurable cognitive decline in some cases, whereas in others cognitive and functional deterioration appears first and mood symptoms follow (Ruthirakuhan et al., 2025). Individuals without depressive symptoms are less affected by this converging set of mechanisms and may be able to mobilize greater neural reserve to compensate and maintain network efficiency. This provides a plausible explanation for the mediating role of depressive symptoms observed in the AD-related MCI group in our data. With respect to AD pathology, depressive symptoms in this group appeared to be more closely linked to the extent of tau pathology than to Aβ deposition. Prior studies have shown that, among amyloid-positive individuals, depressive symptoms predict faster tau progression (Talmasov et al., 2025), whereas in LLD the severity of depressive symptoms and cognitive change is not directly linked to Aβ load (Kim et al., 2024), indicating that tau should be considered when examining depression–AD links (Byeon et al., 2025). Moreover, impairments in attention and executive function tend to be more pronounced than memory deficits in LLD (Invernizzi et al., 2021). All patients in our AD-related MCI group underwent both Aβ and tau PET, and 82% were classified as tau stage C, so the substantial tau pathology in this group may explain why depressive symptoms showed a fully mediating effect specifically on executive function and attention. Consistent with this interpretation, a large prospective cohort has shown that longer depression duration increases dementia incidence, whereas antidepressant treatment can partly reduce this excess risk (Yang et al., 2023). In summary, these findings from PET cohort underscore the clinical importance of monitoring and treating depressive symptoms in AD patients with PET-confirmed tau deposition, even in those with relatively high CR.

## 5 Limitations

Our research has several limitations. First, the CHARLS dataset did not include neuroimaging, which prevented more precise classification of cognitive impairment and increased the likelihood that cases of vascular cognitive impairment were included. Cognitive assessment in CHARLS lacked measures such as the BNT and TMT, commonly used in ADNI for MCI diagnosis (Bondi et al., 2014; Edmonds et al., 2015), therefore, cognitive impairment was defined solely by the education-adjusted MMSE score, which may have introduced bias into subgroup classification. Second, CR was not measured with an identical instrument across the two cohorts. In CHARLS, CR was assembled from education (ordinal highest degree), occupation, and binary leisure/social activities mapped to the three CRIq domains and therefore did not fully reproduce the original CRIq. In the PET cohort, CR was assessed with CRIq and CRI-total values were mostly in the medium to medium–high range. Thus, applying the original five-level CRIq classification would likely have yielded sparse categories and unstable estimates. To keep the two cohorts analytically comparable and to maintain statistical power, we defined high and low CR using cohort-specific median splits. This improves comparability but reduces granularity relative to the five-level CRIq, so absolute CR scores and effect sizes cannot be interpreted as directly comparable across cohorts. Larger studies that administer the same CRIq-based instrument across cohorts will allow finer CR stratification and formal tests of measurement equivalence. Third, although we applied a mediation framework, the cross-sectional design does not allow us to determine the temporal ordering among CR, depressive symptoms, and cognitive performance. Depressive or apathy-like symptoms can also arise later in the AD continuum when cognitive and functional decline become more apparent. The indirect effects reported here should therefore be understood as statistical mediation of observed associations and as one plausible pathway supported by these data, not as evidence of a fixed causal sequence. Longitudinal designs with temporally separated assessments and AD biomarkers will be needed to test directionality more rigorously. Finally, AD pathology in this study was determined only by Aβ PET and tau PET to confirm its presence, without quantitative or regional characterization of deposition patterns, which may nonetheless relate to CR or depressive symptoms. Also, although major cerebrovascular factors and other non-AD etiologies were excluded based on brain MRI, we cannot rule out the presence of vascular pathologies relevant to depressive symptoms, such as moderate WMH or cerebral microbleeds. As the PET cohort sample size was comparatively small, the study was underpowered to detect small-to-moderate effects, and non-significant findings should be interpreted with caution. Future studies should therefore enroll larger cohorts and incorporate both detailed AD pathology measures and cerebrovascular imaging markers to provide more comprehensive analyses.

## 6 Conclusion

Using nationally representative data from CHARLS 2018 and a PET-confirmed clinical cohort, we found that higher CR was associated with lower odds of cognitive impairment and better performance in several cognitive domains. In overall analyses across both cohorts, depressive symptoms partially mediated the associations between CR and cognitive performance. In the AD-related MCI subgroup with substantial tau deposition, depressive symptoms fully mediated the effects of CR on executive function and attention, suggesting that addressing depression is especially important when tau pathology is present. Taken together, these findings indicate that enhancing CR and systematically screening for and treating depressive symptoms may help preserve cognition in middle-aged and older adults.

## Data Availability

The original contributions presented in the study are included in the article/Supplementary material, further inquiries can be directed to the corresponding author.
