# Supplementary material for: Depressive symptoms mediate the relationship between cognitive reserve and cognitive performance in middle-aged and older Chinese adults: evidence from population-based and clinical PET cohorts including cognitively normal and cognitively impaired participants
Source: Front Aging Neurosci. 2026 Jan 5;17:1708268. doi: 10.3389/fnagi.2025.1708268 (PMC12813095; doi:10.3389/fnagi.2025.1708268)
Supplement: Supplementary file 1 [file Table_1.docx]

**Supplementary Materials**

**Supplementary Table S1 Participant inclusion and exclusion process in the two cohorts**

| **Variables** | **Number of participants excluded** | **Number of remaining participants** |
| --- | --- | --- |
| **CHARLS 2018 cohort** | — | n = 20,826 |
| Parkinson Disease | 189 | 20,637 |
| Severe Phrenoblabia | 118 | 20,519 |
| Age | 2,828 | 17,691 |
| Gender | 0 | 17,691 |
| Marital Status | 0 | 17,691 |
| Self-reported Health Status | 962 | 16,729 |
| Self-reported Memory | 96 | 16,633 |
| Self-reported Hearing | 19 | 16,614 |
| Self-reported Eyesight | 35 | 16,579 |
| Residence | 215 | 16,364 |
| Sleeping Hours | 1 | 16,363 |
| Cognitive Impairment Definition | 13,557 | 2,806 |
| Cognitive reserve Definition | 1,089 | 1,717 |
| Self-reported Depression | 81 | 1,636 |
| **PET cohort** |  | n = 134 |
| Less than Middle School Education | 8 | 126 |
| Age | 10 | 116 |
| Prominent Cerebrovascular Abnormalities on Imaging | 5 | 111 |
| Cognitive Impairment of Non-AD Etiology | 4 | 107 |
| Cognitively Normal Subjects with AD Pathology | 2 | 105 |
| Coexisting Severe Psychiatric Disorders | 2 | 103 |
| Contraindications to MR | 3 | 100 |
| Note: For the CHARLS 2018 cohort, exclusions due to Parkinson’s disease, severe phrenoblabia, and age indicate participants who met the prespecified exclusion criteria; all other exclusions reflect missing data in the corresponding variables. For the PET cohort, all exclusions reflect participants who met the exclusion criteria. | | |

**Supplementary Table S2 Definition of cognitive reserve (CR) and related information in the CHARLS 2018 cohort**

| **Contents** | **Definitions** |
| --- | --- |
| **CR domain: Education** |  |
| Educational Level | 1 = No Formal Education (Illiterate)，2 = Did not Finish Primary School，3 = Home School，4 = Elementary School，5 = Middle School，6 = High School, 7 = Vocational School，8 = Two-/Three-Year College/Associate Degree，9 = Four-Year College/Bachelor’s Degree，10 = Master’s Degree，11 = Doctoral Degree/Ph.D. |
| Attended an Educational or Training Course | 0=No，1=Yes |
| **CR domain: Working Activity** |  |
| Occupational Level | 1 = Low skilled manual work, 2 = Skilled manual work, 3 = Skilled non-manual work, 4 = Professional occupation, 5 = Highly responsible or intellectual occupation |
| Vocational and Technical Training | 0=No，1=Yes |
| **CR domain: Leisure Time** |  |
| Interacted With Friends | 0=No，1=Yes |
| Played Ma-jong, Played Chess, Played Cards, or Went to Community Club | 0=No，1=Yes |
| Provided Help to Family, Friends, or Neighbors Who Do Not Live With You | 0=No，1=Yes |
| Went to a Sport, Social, or Other Kind of Club | 0=No，1=Yes |
| Took Part in a Community-Related Organization | 0=No，1=Yes |
| Done Voluntary or Charity work | 0=No，1=Yes |
| Cared for a Sick or Disabled Adult Who Does Not Live With You | 0=No，1=Yes |
| Used the Internet | 0=No，1=Yes |
| Take Care of Grandchildren | 0=No，1=Yes |
| Stock Investment | 0=No，1=Yes |
| Own Other Financial Products | 0=No，1=Yes |

**Supplementary Table S3 Cutoff scores of neuropsychological tests for the operational diagnostic criteria of MCI**

| **Index/ Age group (years)** | | 50 to 59 | 60 to 69 | 70 to 79 |
| --- | --- | --- | --- | --- |
| Memory Function | Delayed Memory | ≤ 5 | ≤ 4 | ≤ 3 |
|  | Recognition Memory | ≤ 20 | ≤ 19 | ≤ 18 |
| Executive Function | TMT A (time, s) | ≥ 80 | ≥ 90 | ≥ 110 |
|  | TMT B (time, s) | ≥ 200 | ≥ 220 | ≥ 260 |
| **Index/ Education group** | | Middle School | High School | College or above |
| Language Function | verbal fluency | ≤ 12 | ≤ 13 | ≤ 14 |
|  | BNT | ≤ 19 | ≤ 21 | ≤ 22 |
| MCI, mild cognitive impairment; TMT, trail making test; BNT, Boston naming test. | | | | |

**Supplementary Table S4 Cross cohort harmonization of study design, measures, and analysis**

| **Aspects** | **CHARLS 2018 cohort (population-based)** | **Clinical PET cohort** |
| --- | --- | --- |
| **Setting and target population** | National population survey of Chinese adults. | Memory clinic and Nuclear Medicine at Nanjing First Hospital. |
| **Age range and stratification** | 50–80 years. Results shown by 50–64, 65–74, ≥75. | Same as the CHARLS 2018 cohort. |
| **CR measurement** | Composite mapped to CRIq domains: Education (highest degree, ordinal), Working Activity (levels 1–5), Leisure/Social items (binary). | CRIq with age-adjusted standardized domain scores (Education years, Working Activity, Leisure Time). |
| **CR operationalization** | Composite from mapped items.  Total CR = sum of components.  High CR vs low CR by cohort median. | Analyses used total score of CRIq after standardization.  High CR vs low CR by cohort median. |
| **Depressive symptoms** | CESD-10. Continuous score reported; threshold >10 used for interpretability. | HAMD-17. Continuous score reported; threshold >7 used for interpretability. |
| **Cognitive status classification** | Cognitively normal vs cognitive impairment  by education-specific MMSE cutoffs. | Cognitively normal vs mild cognitive impairment (MCI) by domain-based operational criteria. |
| **Domain-specific cognition** | Memory (immediate, delayed, recognition); verbal fluency; reasoning ability. | AVLT-H immediate/delayed/recognition for memory; BNT-30 and verbal fluency for language; TMT-A and B for executive function; SDMT and Digit Span tests for attention. |
| **Imaging** | Not applicable. | Amyloid PET and tau PET. |
| **Covariate strategy** | Age and sex a priori. Others added if baseline differences were meaningful or if clinically or causally relevant. When CR is the primary exposure, education is not adjusted. | Same as the CHARLS 2018 cohort. |
| **Cross-cohort interpretation** | Used to establish population-level associations.  Cross-cohort remarks focus on directional consistency rather than metric equivalence. | Used to validate and extend patterns under AD related pathology.  Cross-cohort remarks focus on directional consistency rather than metric equivalence. |
| Abbreviations: PET, positron emission tomography;CR, cognitive reserve; CRIq, Cognitive Reserve Index Questionnaire; CESD-10, 10-items Center for Epidemiologic Studies Depression Scale; HAMD-17, 17-item Hamilton Depression Rating Scale; MMSE, Mini-Mental State Examination; AVLT-H, Auditory Verbal Learning Test, the Huashan version; BNT, Boston Naming Test; TMT, Trail Making Test; SDMT, Symbol Digit Modalities Test; AD, Alzheimer’s disease. | | |

**Supplementary Table S5 Demographic and clinical characteristics of cognitively impaired and cognitively normal participants analyzed for domain-specific cognitive performance in the CHARLS 2018 cohort (n = 916)**

| **Characteristics** | Cognitive Impairment | Cognitively Normal | p value |
| --- | --- | --- | --- |
|  | (n = 397) | (n = 519) |  |
| Age, years (mean±SD) | 69.80±5.09 | 69.43±4.88 | 0.854 |
| Age (n, %) |  |  | 0.960 |
| 50 ~ 64 | 73 (18.4) | 93 (17.9) |  |
| 65 ~ 74 | 248 (62.5) | 329 (63.4) |  |
| ≥ 75 | 76 (19.1) | 97 (18.7) |  |
| Gender (male, %) | 262 (66.0) | 335 (64.5) | 0.700 |
| Education (mean±SD) | 5.02±1.34 | 4.11±1.59 | <0.001**^***^** |
| MMSE (mean±SD) | 22.56±2.81 | 26.94±1.98 | <0.001**^***^** |
| Marital status (n, %) |  |  | 0.712 |
| Married living with spouse | 307 (77.3) | 409 (78.8) |  |
| Married not living spouse | 16 (4.0) | 18 (3.5) |  |
| Separated | 1 (0.3) | 3 (0.6) |  |
| Divorced | 6 (1.5) | 11 (2.1) |  |
| Widowed | 66 (16.6) | 78 (15.0) |  |
| Never Married | 1 (0.3) | 0 (0.0) |  |
| Self-reported Health Status (n, %) |  |  | 0.304 |
| Very Good | 43 (10.8) | 61 (11.8) |  |
| Good | 54 (13.6) | 71 (13.7) |  |
| Fair | 199 (50.1) | 283 (54.5) |  |
| Poor | 75 (18.9) | 83 (16.0) |  |
| Very Poor | 26 (6.5) | 21 (4.0) |  |
| Self-reported Memory Conditions (n, %) |  |  | 0.827 |
| Excellent | 3 (0.8) | 5 (1.0) |  |
| Very Good | 23 (5.8) | 39 (7.5) |  |
| Good | 37 (9.3) | 53 (10.2) |  |
| Fair | 268 (67.5) | 339 (65.3) |  |
| Poor | 66 (16.6) | 83 (16.0) |  |
| Self-reported Hearing (n, %) |  |  | 0.173 |
| Excellent | 4 (1.0) | 7 (1.3) |  |
| Very Good | 59 (14.9) | 91 (17.5) |  |
| Good | 57 (14.4) | 98 (18.9) |  |
| Fair | 230 (57.9) | 275 (53.0) |  |
| Poor | 47 (11.8) | 48 (9.2) |  |
| Self-reported Eyesight (n, %) |  |  | 0.469 |
| Excellent | 5 (1.3) | 13 (2.5) |  |
| Very good | 57 (14.4) | 79 (15.2) |  |
| Good | 65 (16.4) | 93 (17.9) |  |
| Fair | 221 (55.7) | 283 (54.5) |  |
| Poor | 49 (12.3) | 51 (9.8) |  |
| Sleeping hours (n, %) |  |  | 0.120 |
| < 6 hours | 135 (34.0) | 175 (33.7) |  |
| > 8 hours | 30 (7.6) | 23 (4.4) |  |
| 6 ~ 8 hours | 232 (58.4) | 321 (61.8) |  |
| Residence (n, %) |  |  | <0.001**^***^** |
| City | 70 (17.6) | 194 (37.4) |  |
| Semi-rural | 32 (8.1) | 58 (11.2) |  |
| Rural | 294 (74.1) | 265 (51.1) |  |
| Special | 1 (0.3) | 2 (0.4) |  |
| Cognitive reserve (high, %) | 13 (3.3) | 59 (11.4) | <0.001**^***^** |
| Depressive symptoms (present, %) | 143 (36.0) | 125 (24.1) | <0.001**^***^** |

**Supplementary Table S6 Mediation effects of depressive symptoms on the association between cognitive reserve and cognitive performance in subgroups of the CHARLS 2018 cohort**

|  | ACME | | ADE | | Total Effect | | Prop. Mediated | |
| --- | --- | --- | --- | --- | --- | --- | --- | --- |
|  | Estimate value | [95% CI] | Estimate value | [95% CI] | Estimate value | [95% CI] | Estimate value | [95% CI] |
| **Cognitively impaired group (n = 397)** | | | | | | |  | |
| Immediate Memory | 0.1671 | [−0.0434, 0.4844] | **2.1938^**^** | [0.7101, 3.6006] | **2.3608^**^** | [0.8970, 3.7885] | 0.0625 | [−0.0186, 0.2707] |
| Delayed Memory | 0.0677 | [−0.0373, 0.2280] | 0.6182 | [−0.4870, 1.6870] | 0.6858 | [−0.4065, 1.7702] | 0.0584 | [−0.7974, 0.9153] |
| Recognition Memory | 0.0369 | [−0.0734, 0.1924] | 0.7857 | [−0.0636, 1.5958] | 0.8253 | [−0.0052, 1.6432] | 0.0356 | [−0.1834, 0.4523] |
| Episodic Memory | 0.2749 | [−0.0718, 0.8020] | **3.5967^*^** | [0.6858, 6.3756] | **3.8716^**^** | [0.9893, 6.6630] | 0.0618 | [−0.0201, 0.3308] |
| Verbal Fluency | 0.2059 | [−0.0334, 0.5592] | **5.1860^*^** | [1.0294, 9.3665] | **5.3919^*^** | [1.1395, 9.5117] | 0.0343 | [−0.0093, 0.1654] |
| Reasoning Ability | 0.0656 | [−0.0053, 0.1599] | **1.0731^**^** | [0.3351, 1.8170] | **1.1387^**^** | [0.3881, 1.8862] | 0.0536 | [−0.0068, 0.1970] |
| **Cognitively normal group (n = 519)** | | | | | | | | |
| Immediate Memory | 0.0591 | [−1.1124, 0.2334] | **1.4397^*^** | [0.2355, 2.6165] | **1.4988^*^** | [0.2804, 2.6989] | 0.0338 | [−0.1018, 0.2450] |
| Delayed Memory | 0.0182 | [−0.0353, 0.0826] | **0.7396^*^** | [0.1770, 1.2860] | **0.7579^**^** | [0.1976, 1.3091] | 0.0184 | [−0.0563, 0.1649] |
| Phrase Recognition | 0.0004 | [−0.0259, 0.0251] | **0.5098^**^** | [0.2232, 0.7928] | **0.5103^**^** | [0.2232, 0.7871] | 0.0006 | [−0.0618, 0.0575] |
| Episodic Memory | 0.0769 | [−0.1477, 0.3143] | **2.6892^**^** | [0.9154, 4.4424] | **2.7661^**^** | [0.9726, 4.5328] | 0.0231 | [−0.0617, 0.1540] |
| Verbal Fluency | 0.0455 | [−0.0862, 0.1996] | **2.2458^**^** | [0.6200, 3.8287] | **2.2913^**^** | [0.6546, 3.8942] | 0.0158 | [−0.0440, 0.1318] |
| Reasoning Ability | 0.0051 | [−0.0135, 0.0310] | **0.6624^**^** | [0.3347, 0.9838] | **0.6674^**^** | [0.3383, 0.9932] | 0.0037 | [−0.0190, 0.0535] |
| **^*^** *p* <0.05, **^**^** *p* <0.01, **^***^** *p* <0. 001.ACME = average causal mediation effects (indirect effect). ADE = average direct effects. Prop. Mediated describes the proportion of the effects of the independent variable on the dependent variable that goes through the mediator. Estimate value describes the effect size of the mediation effects. ns = not significant. | | | | | | | | |
